# Case-matched comparison of combined phacoemulsification with ab-interno trabeculectomy via Kahook dual blade and trabectome in a Caucasian population

**DOI:** 10.1038/s41598-026-39331-8

**Published:** 2026-03-13

**Authors:** David Kiessling, Hannah Bleiel, Gernot F. Roessler, Thomas S. Dietlein, Randolf A. Widder, Alexandra Lappas

**Affiliations:** 1 Department of Ophthalmology, St. Martinus-Krankenhaus, Gladbacher Str. 26, 40219 Düsseldorf, Germany; 2 Department of Ophthalmology, University Hospital of Cologne, Kerpener Str. 62, 50937 Cologne, Germany; 3 Department of Ophthalmology, RWTH Aachen University, Pauwelstr. 30, 52074 Aachen, Germany

**Keywords:** Glaucoma incisional surgery, MIGS, Trabectome, Kahook dual blade, KDB, Diseases, Medical research

## Abstract

The purpose of this retrospective study was to compare the outcomes after combined phacoemulsification and ab-interno trabeculectomy via Kahook Dual Blade (KDB) and Trabectome, being represented in two groups of patients of Caucasian ethnicity with matched baseline criteria. We included 60 eyes of 49 participants being treated for cataract, of which 30 eyes underwent additional ab-interno trabeculectomy via KDB (Kahook group) and 30 eyes received additional Trabectome surgery (Trabectome group). For this comparative analysis, the Kahook group and Trabectome group were matched at a 1:1-ratio, based on the following criteria: preoperative IOP, maximum known preoperative IOP, preoperative medication score, cup/disc-ratio, follow-up time, best-corrected visual acuity and age. Successful surgery was defined by three scores: IOP at longest follow-up < 21 mmHg (Score A) or < 18 mmHg (Score B) without re-surgery and an IOP reduction > 20% or IOP ≤ 15 mmHg without re-surgery and an IOP reduction ≥ 40% (Score C). Furthermore, we compared postoperative IOP, as well as medication score, and side effects between both groups. Both surgical techniques led to a relative IOP reduction of 29% within their respective groups. Specifically, preoperative IOP decreased from 19.5 ± 5.0 mmHg to 13.8 ± 3.9 mmHg in the Kahook group, and from 19.8 ± 4.5 mmHg to 14.0 ± 3.9 mmHg in the Trabectome group during an average follow-up period of 23–24 months. There was no statistical significant difference noted. Both the KDB and Trabectome yielded similar success rates, according to Score A (67% vs. 70%), Score B (63% vs. 67%) and Score C (33% vs. 23%). There were no severe side effects notes in either group. In conclusion, the KDB and Trabectome showed similar IOP-lowering properties and safety profiles within our two matched groups of Caucasian patients.

## Introduction

During the last decades microinvasive glaucoma surgery (MIGS), which targets the iridocorneal angle, has been used in routine clinical practice as an addition to the established spectrum of traditional filtering surgery. Whilst there is a multitude of surgical techniques, being either trabecular, suprachoroidal or sub-conjunctival, it is the ab-interno approach that these techniques have in common. MIGS procedures have become a popular choice among glaucoma-surgeons for a primary surgical procedure, as they do not require the penetration of the conjunctiva and spare it for secondary filtering surgery if needed. Along with the iStent (Glaucos Corp., Laguna Hills, CA, USA), the Trabectome (Microsurgical Technology, Redmond, WA, USA) and the Kahook Dual Blade (KDB, New World Medical, Rancho Cucamonga, CA, USA) are among the trabecular MIGS procedures with the most extensive period of commercial use and, therefore, with the most robust data^1–9^.

The Trabectome facilitates an electroablation of the trabecular meshwork (TM), aiming to improve outflow through Schlemm’s canal. Additionally, the device incorporates an irrigation and aspiration system for anterior chamber (AC) maintenance^1–3^.

The KDB pursues a similar strategy by excising a strip of trabecular meshwork via a handpiece without being connected to an irrigation and aspiration console^4,5^.

For both the KDB and the Trabectome, a combination with cataract surgery showed a promising IOP-lowering potential, as well as a favorable safety profile^6–9^.

While both techniques share a similar approach, it is still unclear whether one is superior to the other, as results of comparative experimental and clinical studies are somewhat conflicting.

The first publication on the KDB postulated a more complete removal of TM than carried out via Trabectome in human cadaveric corneal rim tissue. Subsequent perfusion studies projected similar IOP-lowering properties for both procedures^10^. However, in a perfused porcine anterior segment model by Dang et al., Trabectome yielded a statistically significantly greater IOP reduction than KDB^11^.

To date, there are two comparative studies evaluating clinical outcomes after combined phacoemulsification and ab-interno trabeculectomy via KDB and Trabectome. Fliney et al. reported that, while KDB achieved a greater reduction of medication burden, the outcomes after Trabectome surgery fulfilled the surgical success criteria more frequently. Within the mixed collective in terms of glaucoma subtype and ethnicity, IOP-lowering was similar among both groups^12^. In a recent study, Mou et al. found no statistical significant difference in IOP reduction achieved by either KDB or Trabectome among Asian patients being diagnosed with open-angle glaucoma (OAG)^13^.

The aim of this study was to compare the outcomes after combined phacoemulsification and ab-interno trabeculectomy via KDB and Trabectome in a Caucasian cohort. The two groups were matched based on a multitude of baseline criteria, in order to deliver a highly balanced comparison.

## Methods

### Study design and patients

This retrospective study was based on the data acquired at two centres (Düsseldorf, Cologne). According to the Ethics Committee of the University of Cologne, an ethical review of the analysis was not required due to the retrospective nature of the study (in accordance with Sect. 15, Paragraph 1 of the Professional Code of Conduct for North Rhine-Westphalian Medical Doctors, Germany). All tenets of the Declaration of Helsinki have been regarded. Informed consent was obtained from all subjects. We reviewed our databases and identified all patients who underwent combined phacoemulsification and ab-interno trabeculectomy either via KDB (Kahook group) or via Trabectome (Trabectome group) from 2010 to 2024. Both datasets originate from two separate eye departments, who are offering the full spectrum of glaucoma care. The procedures were carried out by either of three experienced surgeons (R.W., T.D. and A.L.). We included patients diagnosed with exfoliation glaucoma (XFG), primary open-angle glaucoma (POAG) and primary angle-closure glaucoma (PACG). As tertiary referral centers, all patients are referred to us by ophthalmology practices, having received the diagnosis of manifest glaucoma, based on visual fields and evaluations of the optic nerve head having been carried out during routine-examinations. Patients with concomitant eye disease, such as neovascular glaucoma and uveitis, as well as patients with a history of vitrectomy were excluded. All patients underwent gonioscopy prior to surgery, an open angle, as well as a pigmented TM were a prerequisite for surgery at our centers. The threshold for follow-up was at least 6 months.

The eligible patient groups were matched at a 1:1-ratio, based on the following criteria: preoperative IOP, maximum known preoperative IOP, preoperative medication score, cup/disc-ratio, follow-up time, best-corrected visual acuity (BCVA) and age. In terms of glaucoma subtypes, the ratios of XFG/POAG/PACG were adjusted among the two groups as well.

The IOP was assessed either via Goldmann applanation or iCare rebound tonometry (TAO1i, Tiolat Oy, Helsiki, Finland). According to a recent meta-analysis, the two methods can be considered equivalent, as no significant difference has been observed between them^14^. We collected a maximum of three IOP measurements, prior to the surgery, and averaged them to evaluate the baseline IOP. The medication score comprised the number of IOP-lowering medication classes administered at baseline and follow-up respectively.

### Surgical technique

In Trabectome cases, a standard 1.7–1.8 mm clear cornea incision was made in the temporal periphery. The TM and Schlemm’s canal were visualised using a modified Swan-Jacob goniolens and back-tilting of the microscope. Then, the handpiece was inserted and its footplate penetrated the TM into Schlemm’s canal, electroablation of the trabecular meshwork was carried out within 3 to 4 clock hours. AC depth was maintained by its integrated irrigation and aspiration system.

For subsequent phacoemulsification, the clear cornea incision was then enlarged to an aperture of 2.8 mm, and two paracenteses, at a 90° angle to the tunnel, were made in the peripheral cornea, followed by a bimanual phacoemulsification with posterior chamber and in-the-bag intraocular lens (IOL) implantation.

Ab-interno trabeculectomy via KDB was performed after phacoemulsification and injection of ophthalmic viscosurgical device (OVD) into the AC for stabilization. After TM-visualization, the handpiece was inserted through the tunnel and the blade was driven into the TM with its heel being placed into Schlemm’s canal. Excision was performed within 3 to 4 clock hours as well and the residual TM strip was removed subsequently.

In patients with XFG, additional trabecular aspiration was carried out through the pre-existing paracenteses via a specially designed bent cannula with an outer diameter of 400 μm, linked to an irrigation and aspiration device^15–17^. The procedure was performed on the remaining trabecular meshwork using a suction pressure set to 200 mmHg, in accordance with Jacobi et al.^18^.

Standardised postoperative topical therapy was administered, including steroid eye drops, steroid ointment, antibiotic eye drops, miotic eye drops, as well as the previous topical anti-glaucomatous medication. Further use of IOP-lowering eye drops was based on the discretion of the patient’s referring ophthalmologist^16,17^.

### Outcome measurement

The outcome measurements were chosen according to previous studies by our group^19,20^.

Changes in IOP and medication scores at the longest follow-up examination were the primary clinical endpoints. These outcomes were further defined as success or failure by three separate scores. According to Scores A and B, an absolute IOP < 21 mmHg or < 18 mmHg at the follow-up examination and a postoperative IOP reduction > 20%, respectively, qualified for success. The aforementioned scores were chosen based on the Tube versus Trabeculectomy Study^21^. In contrast, the criteria for Score C were an absolute IOP ≤ 15 mmHg and a postoperative IOP reduction ≥ 40%, based on the criteria of the World Glaucoma Association^22^. Re-surgery was considered a failure in all scores.

### Statistical analyses

We performed the statistical analyses using SPSS (Version 28.0, IBM Corp. Armonk, NY, USA) and the statistical programming language R V3.2.2 (R Foundation for Statistical Computing, Vienna, Austria). We compared the outcome measurements between the Kahook group and the Trabectome group via Mann-Whitney U-test and Fisher**’**s exact test. Moreover, we conducted a log-rank test and visualized the differences using Kaplan-Meier curves, as well as a post hoc power analysis. The threshold for statistical significance was defined as *p* < 0.05.

## Results

For the Kahook group, 30 eyes of 23 patients matched the inclusion criteria. Through 1:1-matching, the Trabectome group was formed, which comprised of 30 eyes of 26 patients. Table 1 summarizes the baseline data.

**Table 1:** Baseline data.

Age (years)	KDB *n* = 30	Trabectome *n* = 30	*p*-value
	73.8 ± 5.0	74.4 ± 6.8	0.70
Caucasian	30/30	30/30	> 0.99
Glaucoma subtype			
XFG	15/30 (50%)	15/30 (50%)	> 0.99
POAG	10/30 (33%)	10/30 (33%)	> 0.99
PACG	5/30 (17%)	5/30 (17%)	> 0.99
Maximum preoperative IOP (mmHg)	28.2 ± 3.9	28.0 ± 5.8	0.89
Actual preoperative IOP (mmHg)	19.5 ± 5.0	19.8 ± 4.5	0.83
Medication score initial	2.3 ± 1.0	2.3 ± 1.0	0.94
Baseline C/D ratio	0.8 ± 0.1	0.8 ± 0.2	0.26
Follow-up time (months)	23.3 ± 24.6	24.4 ± 24.6	0.87
Baseline BCVA (logMAR)	0.23 ± 0.1	0.26 ± 0.1	0.13

*BCVA* best-corrected visual acuity, *C/D* cup/disc, *IOP* intraocular pressure, *KDB* Kahook dual blade, *logMAR* logarithm of the minimum angle of resolution, *PACG* primary angle-closure glaucoma, *POAG* primary open-angle glaucoma, *XFG* exfoliation glaucoma.

Following an average follow-up of 23–24 months, IOP decreased by 29% both in the KDB and the Trabectome group. Specifically, preoperative IOP decreased significantly from 19.5 ± 5.0 mmHg to 13.8 ± 3.9 mmHg in the Kahook group, and from 19.8 ± 4.5 mmHg to 14.0 ± 3.9 mmHg in the Trabectome group. Furthermore, medication scores decreased from 2.3 ± 1.0 mmHg to 1.8 ± 0.7 mmHg in the Kahook group, and from 2.3 ± 1.0 mmHg to 1.7 ± 1.0 mmHg in the Trabectome group. There was no statistical significant difference noted among the two groups (Table 2). A comparison of mean IOP values within follow-up timeframes at 3 months, 6 months, 12 months, and 24 months is given in Table 3.

**Table 2:** Intraocular pressure and medication score at baseline and at follow-up.

IOP preoperative (mmHg)	KDB *n* = 30	Trabectome *n* = 30	*p*-value
	19.5 ± 5.0	19.8 ± 4.5	0.83
IOP postoperative (mmHg)	13.8 ± 3.9	14.0 ± 3.9	0.84
Medication score preoperative	2.3 ± 1.0	2.3 ± 1.0	0.94
Medication score postoperative	1.8 ± 0.7	1.7 ± 1.0	0.49

*IOP* intraocular pressure, *KDB* Kahook dual blade.

**Table 3:** Mean IOP (mmHg) and medication score at baseline and during follow-up.

	KDB				Trabectome			
	*n*		IOP (mmHg)	Med score	*n*		IOP (mmHg)	Med score
Baseline	30		19.5 ± 5.0	2.3 ± 1.0	30		19.8 ± 4.5	2.3 ± 1.0
postOP	30		15.4 ± 7.5	2.3 ± 1.0	30		16.9 ± 7.2	2.3 ± 1.0
3 months	17		13.6 ± 3.3	1.8 ± 0.7	17		12.1 ± 2.3	1.6 ± 0.9
6 months	15		14.0 ± 2.9	1.9 ± 0.6	19		14.1 ± 4.4	1.6 ± 1.1
12 months	16		14.1 ± 2.9	1.9 ± 0.7	14		14.1 ± 3.8	1.5 ± 0.9
24 months	13		14.1 ± 4.8	1.8 ± 0.8	9		14.2 ± 2.8	1.9 ± 0.8

*IOP* intraocular pressure, *KDB* Kahook dual blade.

**Fig. 1:**
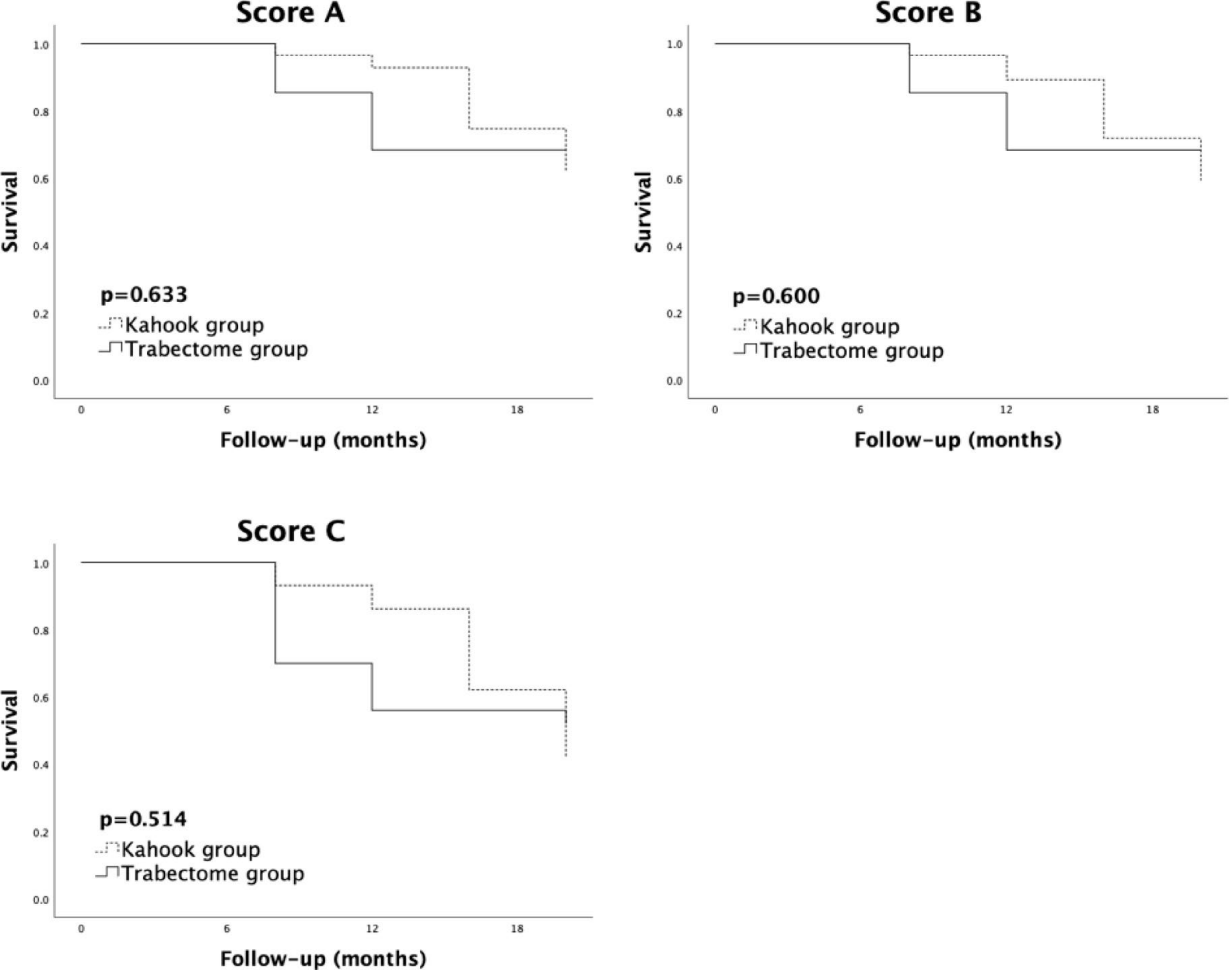
Kaplan-Meier survival curves comparing success rates in the Kahook group and the Trabectome group. Score (**A**) The IOP at follow-up is <21 mmHg, IOP reduction is >20%, and no re-surgery. Score (**B**) The IOP at follow-up is <18 mmHg, IOP reduction is >20%, and no re-surgery. Score (**C**) The IOP at follow-up is ≤ 15 mmHg, IOP reduction is ≥40%, and no re-surgery

The Kaplan-Meier curves showed no statistical significant difference among the two groups terms of success rates when applying Score A (67% vs. 70%), Score B (63% vs. 67%) and Score C (33% vs. 23%) (Fig. 1). Empirical post hoc power analysis showed an 85% power to reveal an inter-group difference of 3 mmHg in IOP reduction, a 51% power to detect a difference of 2 mmHg, and a 17% to detect a difference of 1 mmHg. Furthermore, a 99% power was determined to detect an inter-group difference of 1 in medication score reduction.

Side effects comprised of hyphema, macular edema and anterior chamber fibrin reaction. Hyphema occurred significantly more frequently in the Kahook group (*p* = 0.02). There was no association with anticoagulation use. No severe side effects, such as endophthalmitis, and retinal detachment, were observed in either group (Table 4).

**Table 4:** Complications.

Hyphema	KDB *n* = 30	Trabectome *n* = 30	*p*-value
	7 (23%)	2 (7%)	0.02
Macular edema	2 (7%)	2 (7%)	0.20
Anterior chamber fibrin reaction	0	1 (3%)	0.16
Endophthalmitis, retinal detachment	0	0	> 0.99

*KDB* Kahook dual blade.

## Discussion

In our study, we matched the Kahook and Trabectome group based on a multitude of baseline criteria and found that both surgical techniques yielded a relative IOP reduction of 29%, as well as similar reductions in IOP-lowering medication and success rates during an average follow-up period of 23–24 months. Our results align with those of Mou et al., who came to a similar conclusion within their comparative analysis among Asian patients. Although being methodically solid in analyzing OAG-patients only, one has to take their small sample size, as well as the limited follow-up time of 6 months into account, when interpreting their results^13^.

Previously, Fliney et al. delivered a larger scale study with a more extensive follow-up and a bigger, but also more heterogeneous cohort in terms of glaucoma subtypes and ethnicity. In this study, there was a marked difference in baseline IOP (KDB: 16.9 ± 4.5 mmHg; Trabectome: 18.3 ± 5.9 mmHg). Despite of the absence of a statistical significant difference, it has to be taken into consideration that a low baseline IOP is a major risk factor for surgical failure both for the KDB and Trabectome^23,24^. Therefore, the higher baseline IOP might have contributed to the Trabectome’s higher success rate, whereas KDB led to a greater reduction of medication burden. Although IOP-lowering was ultimately similar among both groups, the authors hypothesized that ethnicity might impact surgical outcomes^12^. For those reasons, we opted to investigate the clinical outcomes in a Caucasian cohort, using a case-matched study design.

For both the KDB and Trabectome we observed a markedly more pronounced IOP-lowering effectiveness in comparison to the aforementioned studies, where less than 20% of IOP reduction have been reported, respectively. This might be explained by the larger proportion of XFG-patients within our cohort, as it has been shown, that both surgical techniques are especially effective in this particular subgroup^25,26^.

Interestingly, hyphema occurred significantly more frequently after KDB surgery. This seems to contradict the findings of Fliney et al., who observed a higher rate of hyphema in the Trabectome group. As in their study, there was no association with the use of anticoagulation agents. While one might expect a greater extent of bleeding in excisional, rather than in electroablative ab-interno trabeculectomy, the authors hypothesized that it might be the use of OVD before the KDB-handpiece is inserted into the AC, that prevents a hyphema from forming through excessive bleeding^12^. Since the extent of OVD-removal via irrigation/aspiration is a potential inter-surgeon variable, this factor might explain the difference between the referred and our study in terms of hyphema occurrence.

In this regard, the Trabectome outcomes might be more reproducible, as the device is equipped with an irrigation and aspiration system and the use of OVD is not necessary for AC maintenance. Also, those properties allow for washing out residual tissue and debris from the treatment site. Remarkably, Seibold et al. postulated that the KDB cut histologically appeared more complete and clean, whereas the Trabectome caused a considerable damage of the adjacent sclera and left residual charred tissue in the TM, as well as debris within the more distal collector channels^10^. On the contrary, Dang et al. reported no collateral damages of any kind and a larger proportion of full-thickness TM ablation via Trabectome^11^. Other authors have discussed that excisional ab-interno trabeculectomy, when not initiated reliably, might inadvertently result in remaining TM lips and hypothesized that those might reapproximate with time^27^.

However, those histological differences do not seem to translate into major clinical implications. As a randomized controlled trial (RCT) found no added benefit of performing KDB over phacoemulsification^28^, one might argue that the inclusion of eyes having received solely phacoemulsification for an additional control group would have been appropriate. We opted against this, as postulating a superiority of MIGS combined with phacoemulsification vs. phacoemulsification-only was not the intention of our study. Rather, the comparison between KDB and Trabectome, as additions to phacoemulsification respectively, was the main objective of this study. Further RCTs are desirable to examine the significance of MIGS combined with phacoemulsification vs. phacoemulsification-only.

One might argue that our study collective is somewhat mixed, as we included patients diagnosed with XFG, POAG, and PACG. We opted to do so, as for both the KDB and Trabectome there is evidence for effectiveness across all of those glaucoma subtypes^9,23^. We acknowledge that the clinical validity of our study might arguably have been impeded by variations in IOP and medication-use during heterogeneous follow-up time points and the conflation of those in order to form a clinical endpoint. Given that those are inherent issues of clinical glaucoma studies, we chose two approaches in order to improve validity. Firstly, we matched the two groups according to the follow-up time. Secondly, we opted for a Kaplan-Meier analysis to compare the clinical endpoints, being a standard asset to evaluate endpoints with varying follow-up time intervals^29^. The retrospective study design as well as the inherent limited sample size were further limitations of our study. An aspect, where the surgical approach might arguably differ is, whether to carry out phacoemulsification or Trabectome surgery first. We opted for the latter, as the Trabectome device requires a smaller incision, which can be easily enlarged for subsequent phacoemulsification. We have made the experience that there is a higher immanent risk of intraoperative shallowing of the anterior chamber, when Trabectome surgery is be carried out through the standard 2.8 mm clear cornea incision after phacoemulsification.

Although further comparative studies on long-term efficacy are desirable, ideally RCTs on this matter, we draw the conclusion that the KDB and Trabectome showed similar IOP-lowering properties and safety profiles. Herein, this was validated via an analysis of two matched groups of Caucasian patients. While surgery time for experienced surgeons, as well as the cost for the disposable handpieces are quite similar respectively, the Trabectome system requires a console, therefore adding a considerable upfront cost. Also, the device requires additional space in the operating room. However, the irrigation/aspiration function for AC maintenance and visualization of the TM might lead to a shorter learning curve for Trabectome surgery when compared with the KDB.

As these geographical, economical or logistical factors might influence the choice of either device, we provided further evidence that both procedures can be considered as equally good options.

## Data Availability

The datasets generated during and/or analysed during the current study are available from the corresponding author on reasonable request.
